# Epidemiological and clinical analysis of 291 children diagnosed with *Chlamydia pneumoniae* pneumonia: a 10-year retrospective study in Shijiazhuang, China

**DOI:** 10.3389/fped.2025.1681564

**Published:** 2025-10-24

**Authors:** Ran Ma, Yingqian Zhang, Yingxue Wang, Yu Liu, Chunxiao Ba, Mengqiao Zhang

**Affiliations:** ^1^ Graduate School, Hebei Medical University, Shijiazhuang, Hebei, China; ^2^ Department No. 3 of Respiratory Medicine, Hebei Children’s Hospital, Shijiazhuang, Hebei, China; ^3^ Hebei Clinical Medicine Research Center for Children's Health and Diseases, Shijiazhuang, Hebei, China; ^4^ Hebei North University, Zhangjiakou, China

**Keywords:** children, *Chlamydia pneumoniae* pneumonia, clinical features, epidemiological characteristics, treatment

## Abstract

**Objective:**

This study aims to systematically analyze the epidemiological and clinical features of *Chlamydia pneumoniae* pneumonia (CPP) in children with community-acquired pneumonia, providing an evidence-based foundation for clinical diagnosis and treatment strategies.

**Methods:**

A retrospective analysis was conducted using clinical data from 291 children diagnosed with CPP who had been admitted to Hebei Children's Hospital between January 2015 and May 2025.

**Results:**

The sex distribution showed a male-to-female ratio of 187:104. The mean age was 8.12 years (range: 1 month–16 years), with an average hospital stay of 7.52 days and a mean total disease duration of 14.81 days. Annual incidence rates exhibited a progressively increasing trend, with peak seasonal occurrence observed during the winter and spring months. Age group analysis revealed the highest prevalence among children aged 7–16 years (198 cases, 68.04%), followed by infants aged 1 month–1 year (65 cases, 22.34%). Cough was the predominant clinical manifestation (141 patients, 98.60%), followed by fever (44.80% of patients; median peak temperature: 38.20 °C). Physical examination revealed pulmonary rales in 288 patients (98.97%). Laboratory findings indicated elevated white blood cell counts with neutrophil predominance, whereas C-reactive protein levels were normal or only mildly elevated. Some patients had abnormalities in coagulation profiles, myocardial enzyme levels, and immune function parameters. Polymicrobial infections were detected in 177 patients (60.82%), with rhinovirus, *Haemophilus influenzae*, and *Streptococcus pneumoniae* identified as the primary co-detected pathogens. Age-specific mixed infection patterns were noted. Imaging studies revealed bilateral lung involvement in approximately half of the patients. Bronchoscopic evaluation demonstrated bronchial mucositis with flocculent secretions. Cytological examination of alveolar lavage fluid showed a predominance of neutrophils, monocytes, and phagocytes. Antimicrobial therapy included monotherapy with doxycycline (30.58%), azithromycin (23.71%), and erythromycin (11.34%). During hospitalization, the antibiotic regimen was switched for seven patients due to intolerance or inadequate response to the initial therapy. All patients achieved complete recovery at discharge, and no deaths were recorded.

**Conclusions:**

This study demonstrates that CPP exhibits distinct seasonal and age-related distribution patterns. As the clinical manifestations and routine laboratory markers offer limited diagnostic sensitivity, confirmatory testing is necessary. Our findings support PCR as a valuable diagnostic tool for this purpose.

## 1 Introduction

*Chlamydia pneumoniae* (CP) is an important pathogen in pediatric community-acquired pneumonia (CAP), accounting for approximately 1%–2% of childhood CAP cases (1, 2), with a reported fatality rate ranging from 3.7%–15% (3). Among children with CAP who have underlying conditions (e.g., respiratory or cardiac malformations, acute lymphoblastic leukemia, or hypogammaglobulinemia), the detection rate of CP is significantly higher (3). This elevated prevalence correlates with a greater risk of severe infections and life-threatening complications, including septic shock and acute respiratory distress syndrome (3–5). The incubation period for CP averages 3–4 weeks. Early infection often presents as mild, self-limiting respiratory illnesses—such as pharyngitis, bronchitis, or sinusitis—or as asymptomatic carriage, typically yielding a favorable prognosis (4). However, emerging evidence links CP infection to chronic obstructive pulmonary disease, asthma, arthritis, cardiovascular disorders, and certain neurological conditions (6, 7). A recent systematic review suggested that CP infection increases the risk of lung cancer (odds ratio: 1.48–1.60) (8), indicating broader clinical implications beyond conventional understanding.

CP exhibits a unique biphasic developmental cycle, disseminating through peripheral blood monocytes to extrapulmonary tissues and causing multisystem damage (9). Epidemiological studies reveal distinct population and seasonal distribution patterns: higher prevalence in infants and school-aged children, peak incidence during autumn and winter months, and cyclical epidemics approximately every four years (7, 10). The clinical manifestations of pediatric *Chlamydia pneumoniae* pneumonia (CPP) are frequently atypical, characterized primarily by persistent fever and cough, whereas laboratory markers and pulmonary imaging findings lack diagnostic specificity. As an obligate intracellular Gram-negative bacterium, CP requires highly sensitive polymerase chain reaction (PCR) assays for definitive identification (11, 12). Delayed or missed diagnosis often results in progressive lung injury, complicating therapeutic management.

Current literature predominantly addresses adult populations or sporadic pediatric cases, with limited systematic analyses of the clinical features and treatment strategies for CPP in children. While macrolides remain the first-line therapeutic agents, emerging concerns regarding antibiotic resistance, corticosteroid use, and vaccine development warrant urgent attention.

Therefore, a comprehensive analysis of CPP epidemiology, clinical characteristics, and diagnostic and therapeutic paradigms is essential for optimizing treatment protocols, improving prognostic outcomes, and enhancing pediatric quality of life. This retrospective study investigated clinical data from CPP patients at Hebei Children's Hospital, comprehensively assessing their epidemiological patterns, clinical manifestations, laboratory and imaging results, treatment modalities, and prognostic outcomes. The findings provide evidence-based guidance for improving early recognition of CPP and advancing precision medicine approaches in pediatric practice.

## 2 Materials and methods

### 2.1 Study subjects

**Figure 1:**
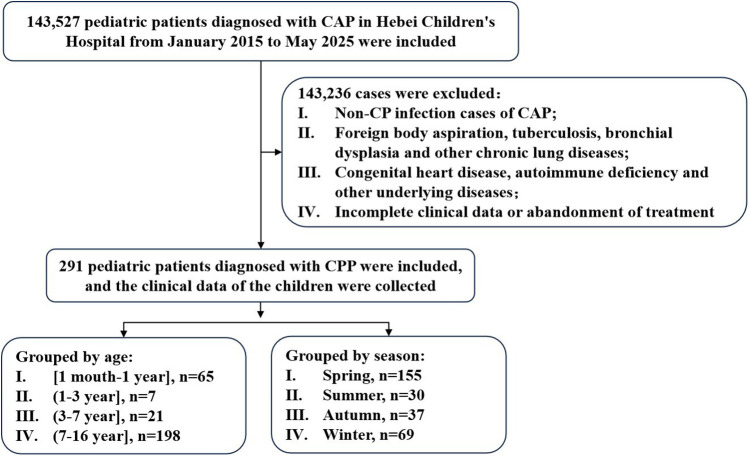
Flow diagram of the study. CP, *Chlamydia pneumoniae*; CAP, community-acquired pneumonia; CPP, *Chlamydia pneumoniae* pneumonia.

This retrospective analysis was conducted on clinical data from 291 children diagnosed with CPP who were admitted to Hebei Children's Hospital between January 2015 and May 2025 (Figure 1). The analysis encompassed demographic characteristics, seasonal distribution patterns, clinical manifestations, laboratory findings, imaging results, mixed infection profiles, treatment modalities, and clinical outcomes.

The diagnostic criteria for CPP are based on the “Guidelines for the Management of Community-Acquired Pneumonia in Children (2024 Revision)” (13). All participants met the following inclusion criteria: (i) presence of pneumonia-related clinical manifestations (e.g., fever, cough); (ii) auscultatory findings of pulmonary rales; (iii) inflammatory lesions on chest x-ray or computed tomography (CT), defined as one or more of the following: bronchial wall thickening, peribronchial infiltration, centrilobular nodules, ground-glass opacities, or consolidation. All imaging studies were reviewed and confirmed by pediatric radiologists; and (iv) laboratory confirmation of CP DNA or RNA positivity. The exclusion criteria were as follows: (i) chronic lung diseases such as foreign body aspiration, tuberculosis, and bronchopulmonal dysplasia; (ii) underlying conditions, such as congenital heart disease or immune deficiencies; and (iii) children with incomplete clinical data, those admitted but later discharged against medical advice, or those with treatment refusal.

### 2.2 Clinical data collection

The following clinical data were systematically collected: (1) demographic data and clinical manifestations; (2) laboratory indicators; (3) etiological results; (4) imaging characteristics; (5) bronchoscopic manifestations; and (6) therapeutic interventions and clinical outcomes.

### 2.3 Etiological testing

At admission, nasopharyngeal swabs were collected from 281 children and analyzed using a multiplex PCR-based respiratory pathogen panel (13-Pathogen Detection Kit; Ningbo Haiershi Gene Technology Co., Ltd., China; Supplementary Table S1). The assay was performed on a GenomeLab™ GeXP genetic analysis system (AB SCIEX, USA) using capillary electrophoresis, with results interpreted by the system's built-in software in accordance with standard operating procedures. Targeted next-generation sequencing (tNGS) was conducted on bronchoalveolar lavage (BAL) fluid samples obtained from ten children hospitalized with severe community-acquired pneumonia (CAP) who met indications for bronchoscopy. These samples were sent directly for tNGS without prior qPCR testing. Sequencing was conducted by Huada Medical Laboratory (Shijiazhuang, China; Supplementary Table S2) after obtaining informed consent from legal guardians and in accordance with relevant clinical guidelines (13, 14). Detailed protocols for library preparation, sequencing, and bioinformatic analysis are provided in the Supplementary Materials (Supplementary_mNGS).

### 2.4 Grouping

Patients were divided into four age groups: [1 month–1 year], [1–3 years], [3–7 years], and [7–16 years]. They were also categorized by season: spring (March–May), summer (June–August), autumn (September–November), and winter (December–February).

### 2.5 Statistical analysis

IBM SPSS Statistics (version 27.0; IBM Corp., Armonk, NY, USA) was used for all statistical analyses. Continuous variables are presented as mean with 95% confidence interval (CI). Differences among groups were compared using one-way analysis of variance (ANOVA), followed by Tukey's Honest Significant Difference (HSD) test for pairwise comparisons. Categorical variables are expressed as counts (percentages) and were compared using the chi-square (*χ*²) test or Fisher's exact test. A two-sided *p*-value < 0.05 was considered statistically significant. Graphs were generated using GraphPad Prism (version 9.5; GraphPad Software, San Diego, CA, USA).

## 3 Results

### 3.1 Study population and clinical features

**Figure 2:**
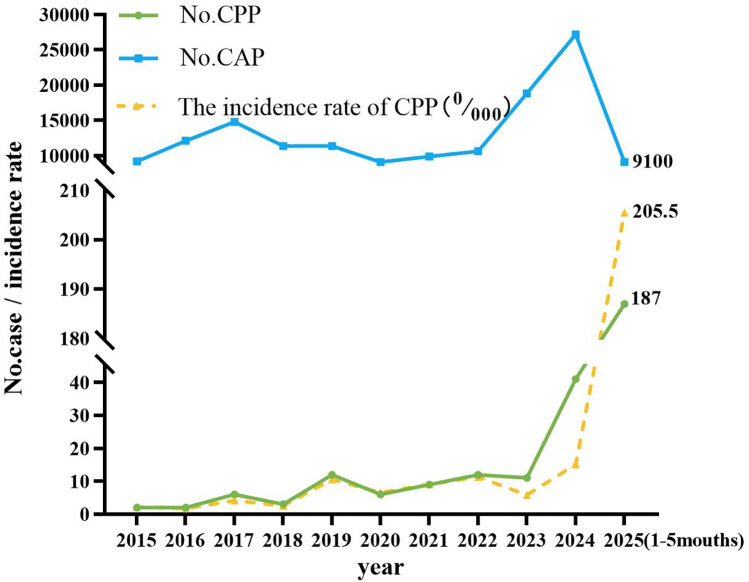
The incidence rate of CPP in pediatric patients in different months. The *x*-axis denotes the calendar year, whereas the *y*-axis quantifies either the absolute case count or the prevalence rate of pediatric patients diagnosed with CPP. CAP, community-acquired pneumonia; CPP, *Chlamydia pneumoniae* pneumonia.

**Figure 3:**
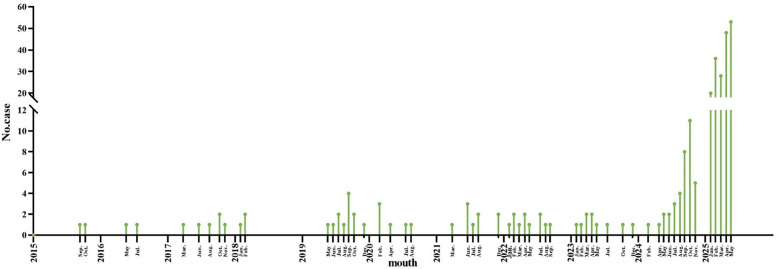
Trend of the incidence of pediatric CPP patients in different months. The *x*-axis represents the mouth, and the *y*-axis represents the number of CPP cases. CPP, *Chlamydia pneumoniae* pneumonia.

The annual incidence of CPP has shown a progressively increasing trend (Figures 2, 3). Seasonal distribution peaked during the winter and spring: 155 cases (53.26%) occurred in spring (March–May), 30 cases (10.31%) in summer (June–August), 37 cases (12.71%) in autumn (September–November), and 69 cases (23.71%) in winter (December–February) (Table 1). Among the 291 pediatric patients, the male-to-female ratio was 187:104. The mean hospitalization duration was 7.52 days, and the mean total disease course was 14.81 days. The average age was 8.12 days (range: 1 month–16 years). Age distribution analysis revealed the highest prevalence in the 7–16–year age group (198 cases, 68.04%), followed by the 1-month–1-year age group (65 cases, 22.34%).

**Table 1:** The demographic and clinical characteristics.

Variables		*N* = 291	Variables	*N* = 291
Sex, *n* (%)	Boys	187 (64.30%)	Hospitalization (d)	7.52 (7.12–7.92)
	Girls	104 (35.70%)	Total diseasecourse (d)	14.81 (13.52–16.10)
Age (year)		8.12 (7.54–8.70)	Fever, *n* (%)	133 (45.70%)
Age (year), *n* (%)	[1 mouth-1]	65 (22.34%)	Peak temperature (*n* = 133) (°C)	38.44 (38.34–38.55)
	[1–3]	7 (2.41%)	Cough, *n* (%)	288 (98.97%)
	[3 -7]	21 (7.22%)	Shortness of breath, *n* (%)	31 (10.65%)
	[7–16]	198 (68.04%)	Rale, *n* (%)	140 (48.11%)
Season, *n* (%)	Spring	155 (53.26%)	Respiratory failure, *n* (%)	2 (0.69%)
	Summer	30 (10.31%)	Myocardial damage, *n* (%)	6 (2.06%)
	Autumn	37 (12.71%)	Rash, *n* (%)	6 (2.06%)
	Winter	69 (23.71%)	Gastrointestinal symptoms, *n* (%)	15 (5.15%)
			Thoracodynia, *n* (%)	1(0.34%)

Continuous variables are presented as the mean [95% confidence interval (CI)].

The most common clinical manifestation was cough (288 patients, 98.97%), followed by fever (133 patients, 45.70%), with a mean peak temperature of 38.4 °C. Other pulmonary findings included rales (140, 48.11%), shortness of breath (31, 10.65%), and respiratory failure (2, 0.69%). Extrapulmonary manifestations (Table 1) were observed in a subset of patients, including myocardial damage (6, 2.06%), rash (6, 2.06%), gastrointestinal symptoms such as abdominal bloating, vomiting, and diarrhea (15, 5.15%), and thoracodynia (1, 0.34%).

### 3.2 Laboratory indicators

The laboratory profiles of the 291 enrolled patients are summarized in Table 2. The mean white blood cell (WBC) count was 14.39 × 10⁹/L (95% CI: 9.21–19.57). Differential counts showed a mean neutrophil percentage (NEUT%) of 57.65% (95% CI: 55.90–59.41) and a mean lymphocyte percentage (LYMPH%) of 31.21% (95% CI: 29.71–32.72). The mean hemoglobin (Hb) level was 128.76 g/L (95% CI: 126.27–131.24), and the mean platelet (PLT) count was 339.37 × 10⁹/L (95% CI: 326.77–351.97).

**Table 2:** Laboratory indicators.

Variables	*N* = 291	Variables	*N* = 291, *n*(%)
WBC (10^9^/L)	14.39 (9.21–19.57)	Lactate dehydrogenase (U/L)	243.51 (236.49–250.53)
NEUT (%)	57.65 (55.90–59.41)	Creatine kinase isoenzyme (ng/ml)	2.67 (2.54–2.79)
LYMPH (%)	31.21 (29.71–32.72)	Fibrinogen (g/L)	3.00 (2.89–3.11)
Hb (10^9^/L)	128.76 (126.27–131.24)	Abnormal immune function	64 (21.99%)
PLT (10^9^/L)	339.37 (326.77–351.97)	Abnormal liver function	83 (28.52%)
CRP (mg/L)	10.74 (8.99–12.49)	Abnormal kidney function	36 (12.37%)

Continuous variables are presented as the mean [95% confidence interval (CI)]. Details of the laboratory panels (liver function, renal function, cardiac enzyme and immune function) are provided in the Supplementary Materials (see “Supplementary_ Laboratory Panels Summary” for details).

Regarding inflammatory and cardiac markers, the mean C-reactive protein (CRP) level was 10.74 mg/L (95% CI: 8.99–12.49). The mean lactate dehydrogenase (LDH) level was 243.51 U/L (95% CI: 236.49–250.53), and the mean creatine kinase isoenzyme (CK-MB) level was 2.67 ng/ml (95% CI: 2.54–2.79). The mean fibrinogen level was 3.00 g/L (95% CI: 2.89–3.11).

The prevalence of organ function abnormalities was also assessed. Abnormal liver function was observed in 83 patients (28.52%), abnormal immune function in 64 patients (21.99%), and abnormal kidney function in 36 patients (12.37%).

### 3.3 Etiological results

Etiological testing was performed on all 291 patients. The overall detection rate of CP was 100%, with 114 cases (39.18%) identified as CP monoinfection, and 177 cases (60.82%) as CP mixed infections with other pathogens. Among co-detected viruses, rhinovirus was the most prevalent (58 cases, 19.93%), followed by *Human metapneumovirus* (11 cases, 3.78%), *Cytomegalovirus* (8 cases, 2.75%), *Respiratory syncytial virus* (7 cases, 2.41%), and both *Adenovirus* and *Influenza A virus* (5 cases each, 1.72%).

Among bacterial co-infections, *Haemophilus influenzae* and *Streptococcus pneumoniae* (SP) were the most common, each detected in 53 cases (18.21%). This was followed by *Mycoplasma pneumoniae* (39 cases, 13.40%), *Staphylococcus aureus* (9 cases, 3.09%), and *Klebsiella* species (6 cases, 2.06%) (Table 3).

**Table 3:** Etiological results.

Variables	*N* = 291, *n* (%)	Variables	*N* = 291, *n* (%)
CP-Single infection	114 (39.18%)	*Influenza A virus*	5 (1.72%)
CP-mixed infection	177 (60.82%)	*Haemophilus influenzae*	53 (18.21%)
*Rhinovirus*	58 (19.93%)	*Streptococcus pneumoniae*	53 (18.21%)
*Human metapneumovirus*	11 (3.78%)	*Staphylococcus aureus*	9 (3.09%)
*Cytomegalovirus*	8 (2.75%)	*Klebsiella*	6 (2.06%)
*Respiratory syncytial virus*	7 (2.41%)	*Mycoplasma pneumoniae*	39 (13.40%)
*Adenovirus*	5 (1.72%)		

### 3.4 Imaging and bronchoscopic findings

**Figure 4:**
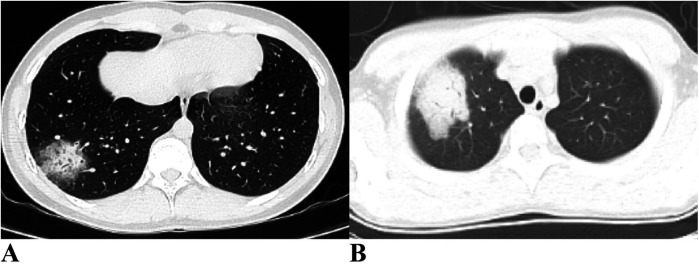
Ct imaging features of the lungs in children with CPP. **(A)** Halo sign and air bronchogram sign are evident in the right lung. **(B)** Consolidation is observed in the right lung. CPP, *Chlamydia pneumoniae* pneumonia.

**Figure 5:**
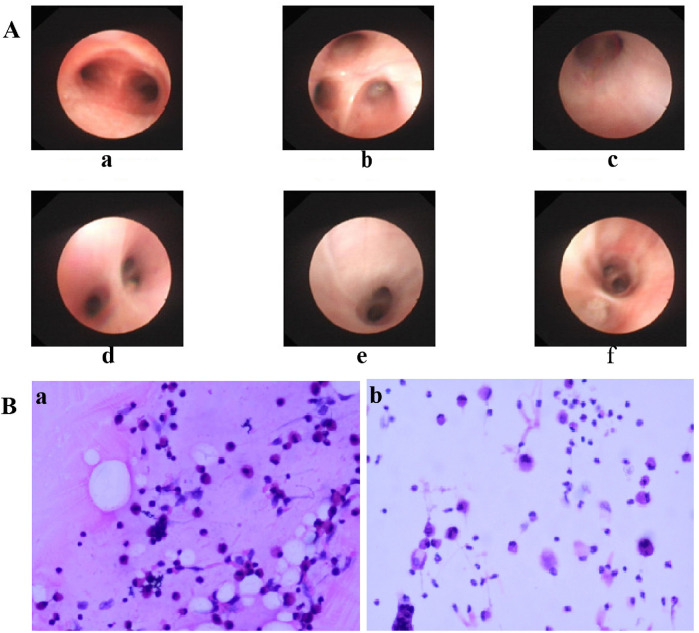
**(A)** Bronchoscopic manifestations of children with CPP. Rough and slightly pale mucous were seen in the upper lobe of the right lung B1(c), B2(d), B3(e) and the lower lobe of the right lung B7(f), and a small amount of white flocculent secretions were seen adhering to the bronchial walls. a: tracheal carina; b: right upper lobe. **(B)** Cytopathologic Findings in Alveolar Lavage Fluid of Pediatric CPP (HE Staining). Patient a: Macrophages/Monocytes 38%, Lymphocytes 25%, Neutrophils 35%, Eosinophils 0%, Bronchial Ciliated Cells 2%, Squamous Epithelial Cells 0%. Patient b: Macrophages/Monocytes 28%, Lymphocytes 5%, Neutrophils 57%, Eosinophils 0%, Bronchial Ciliated Cells 6%, Squamous Epithelial Cells 4%. CPP, *Chlamydia pneumoniae* pneumonia.

As shown in Table 4, approximately half of the pediatric patients exhibited involvement of two or more lung lobes. Lung imaging findings frequently include the halo sign, bronchial inflation sign, or consolidation (Figure 4). Flexible bronchoscopy revealed bronchial mucositis with flocculent secretions in the examined patients (Figure 5A). Cytological analysis of BAL fluid revealed a predominance of neutrophils, followed by monocytes and alveolar macrophages (Figure 5B).

**Table 4:** Imaging characteristics.

Variables	*N* = 291, *n*(%)	Variables	*N* = 291, *n*(%)
Pulmonary radiological features		Pleural thickening	9 (3.09%)
Bronchial pneumonia	242 (83.16%)	Pleural adhesions	2 (0.69%)
≥2 lung lobes	171 (58.76%)	Emphysema	1 (0.34%)
Consolidation	105 (36.08%)	Pulmonary cavity	1 (0.34%)
Pleural effusion	12 (4.12%)	Bronchoscopy with alveolar lavage	67 (23.02%)
Atelectasis	2 (0.69%)	Mucosal congestion and edema	66 (22.68%)
Lung abscess	3 (1.03%)	Postoperative laryngeal edema	1 (0.34%)
Bronchiectasis	5 (1.72%)	Pericardial effusion	4 (1.37%)

### 3.5 Treatment

All patients received treatment after hospitalization. Initial antimicrobial strategies and subsequent adjustments are summarized as follows. Monotherapy was initiated with doxycycline (89 patients, 30.58%), azithromycin (69, 23.71%), erythromycin (33, 11.34%), minocycline (16, 5.50%), and other agents (8, 2.74%). During treatment, two patients were switched to azithromycin for better tolerability, and five required escalation to minocycline or doxycycline due to azithromycin failure. Seventeen patients (5.84%) received corticosteroids for severe inflammation (Table 5). Sixty-nine patients (23.71%) received only inpatient supportive care due to confirmed antibiotic use prior to admission. All patients achieved complete clinical recovery before discharge. Recovery was uneventful, with no ICU admissions, mechanical ventilation, or other complications, and no mortality was reported.

**Table 5:** Treatments.

Variables	*N* = 291, *n* (%)	Variables	*N* = 291, *n* (%)
Antibiotics		Clarithromycin—Azithromycin	1 (0.34%)
Erythromycin	33 (11.34%)	Erythromycin—Azithromycin	1 (0.34%)
Azithromycin	69 (23.71%)	Azithromycin—Doxycycline	3 (1.03%)
Clarithromycin	3 (1.03%)	Azithromycin—Minocycline	2 (0.69%)
Acetylmidecamycin	4 (1.37%)	Intravenous Immunoglobulin	2 (0.69%)
Acetylkitasamycin	1 (0.34%)	Glucocorticoids	28 (9.62%)
Doxycycline	89 (30.58%)	Methylprednisolone	25 (8.59%)
Minocycline	16 (5.50%)	Dexamethasone	3 (1.03%)
		Lung physical therapy	4 (1.37%)

### 3.6 Variations in clinical presentation, laboratory findings, and imaging features across age groups

As shown as Table 6, the cohort was stratified into four age groups for comparative analysis: (1 month–1 year) (Group a, *n* = 65), (1–3 years) (Group b, *n* = 7), (3–7 years) (Group c, *n* = 21), and (7–16 years) (Group d, *n* = 198). Significant variations in clinical presentation, laboratory parameters, and radiological features were observed across these groups.

**Table 6:** Comparison of clinical characteristics, laboratory and imaging results among different age groups.

Variables	[1 mouth-1 year](a), *N* = 65	(1–3 year](b), *N* = 7	(3–7 year](c), *N* = 21	(7–16 year](d), *N* = 198	*P* value
Clinical characteristic					
Hospitalization(d)	8.97 (7.66–10.28)	5.57 (2.86–8.29)	6.76 (5.78–7.75)	7.19 (6.83–7.55)	***P*_ac_** **=** **0.002**
					***P*_ad_** **<** **0.001**
Diseasecourse (d)	19.06 (15.80–22.33)	12.14 (6.71–17.57)	15.00 (9.47–20.53)	13.48 (12.06–14.91)	***P*_ad_** **<** **0.001**
Fever, *n* (%)	6 (9.23%)	3 (42.86%)	13 (61.90%)	111 (56.06%)	***P*** **<** **0.001**
Cough, *n* (%)	65 (100.00%)	7 (100.00%)	21 (100.00%)	195 (98.48%)	-
Laboratory test					
WBC (10^9^/L)	12.29 (11.11–13.46)	12.17 (7.62–16.73)	9.53 (8.31–10.76)	15.67 (8.05–23.67)	*P* = 0.905
NEUT (%)	44.04 (39.76–48.32)	48.97 (35.39–62.55)	58.48 (53.45–63.51)	62.34 (60.68–64.00)	***P*_ac_** **<** **0.001**
					***P*_ad_** **<** **0.001**
					***P*_bd_** **=** **0.009**
LYMPH (%)	40.97 (36.81–45.13)	40.13 (25.52–54.73)	31.21 (25.99–36.44)	27.70 (26.35–29.04)	***P*_ac_** **=** **0.001**
					***P*_ad_** **<** **0.001**
					***P*_bd_** **=** **0.007**
Hb (10^9^/L)	113.68 (109.02–118.34)	127.10 (118.52–135.69)	129.25 (126.37–132.14)	133.71 (130.70–136.73)	***P*_ac_** **=** **0.002**
					***P*_ad_** **<** **0.001**
PLT (10^9^/L)	391.40 (356.94–425.86)	333.01 (246.13–419.89)	336.46 (288.17–384.75)	322.82 (309.75–335.88)	***P*_ac_** **=** **0.004**
					***P*_ad_** **<** **0.001**
CRP (mg/L)	3.65 (2.06–5.23)	14.60 (4.33–33.53)	7.56 (2.47–12.66)	13.27 (10.94–15.59)	***P*_ad_** **<** **0.001**
LDH (U/L)	289.34 (274.68–304.00)	268.29 (225.20–311.37)	252.33 (236.92–267.74)	226.66 (218.82–234.49)	***P*_ac_** **=** **0.008**
					***P*_ad_** **<** **0.001**
					***P*_cd_** **=** **0.044**
CK-MB (ng/ml)	3.43 (3.11–3.75)	2.93 (0.80–5.05)	2.33 (2.11–2.55)	2.45 (2.33–2.57)	***P*_ac_** **<** **0.001**
					***P*_ad_** **<** **0.001**
Fibrinogen (g/L)	2.23 (2.08–2.39)	2.66 (1.56–3.75)	3.02 (2.70–3.33)	3.26 (3.13–3.39)	***P*_ac_** **<** **0.001**
					***P*_ad_** **<** **0.001**
Pulmonary radiological features, *n*(%)					
Consolidation	2 (3.08%)	1 (14.29%)	11 (52.38%)	91 (45.96%)	***P*** **<** **0.001**
Pleural effusion	1 (1.54%)	0 (0.00%)	1 (4.76%)	10 (5.05%)	*P* = 0.596
Codetected pathogens	*Cytomegalovirus* 8 (12.31%); *Rhinovirus* 5 (7.69%); *Streptococcus pneumoniae* 4 (6.15%)	*Rhinovirus* 2 (28.57%); *Haemophilus influenzae* 2 (28.57%); *Adenovirus* 2 (28.57%)	*Mycoplasma pneumoniae* 9 (42.86%); *Haemophilus influenzae* 7 (33.33%); *Streptococcus pneumoniae* 6 (28.57%)	*Rhinovirus* 46 (23.23%); *Haemophilus influenzae* 44(22.22%); *Streptococcus pneumoniae* 43(21.72%)	

Continuous variables are presented as the mean [95% confidence interval (CI)].

Bold values indicate significant at *P* < 0.05.

Clinically, the duration of hospitalization and the total disease course were significantly longer in the youngest infants (Group a) compared to older children (Groups c and d). The prevalence of fever increased markedly with age, occurring in only 9.23% of Group a compared to 61.90% in Group c and 56.06% in Group d (*P* < 0.001). Cough was a ubiquitous symptom across all groups. Laboratory findings revealed distinct age-related patterns. The neutrophil percentage (NEUT%) increased significantly with age, whereas the lymphocyte percentage (LYMPH%) showed a converse, decreasing trend. Platelet (PLT) counts were highest in Group a and declined in older children. Several inflammatory and tissue damage markers also varied significantly: C-reactive protein (CRP) levels were higher in Group a than in Group d (*P* < 0.001), while lactate dehydrogenase (LDH) and CK-MB levels decreased with advancing age. Radiological assessment indicated that pulmonary consolidation was rare in Group a (3.08%) but significantly more prevalent in older children, affecting 52.38% of Group c and 45.96% of Group d (*P* < 0.001). The profile of co-detected respiratory pathogens also differed: *Mycoplasma pneumoniae* and *Haemophilus influenzae* were most common in Group c, and *rhinovirus* and *Haemophilus influenzae* were most frequently detected in Group d.

## 4 Discussion

As a significant cause of CAP, CP exhibits a well-documented cyclical pattern, with major epidemic peaks recurring approximately every four years (3). This periodicity reflects waning population immunity, which allows renewed transmission once susceptibility reaches a critical threshold. Seasonal factors such as cold-weather indoor crowding and high-contact settings like schools further amplify transmission, driving recurrent epidemic waves. Our findings are consistent with this model but indicate that it was significantly influenced by recent events. The historically reported prevalence of 1%-2% among pediatric CAP cases (1, 2) contrasts with our observed endemic prevalence of below 0.5% between 2015 and 2024. We propose that this period represents an extended trough, prolonged by the suppression of CP circulation due to COVID-19 non-pharmaceutical interventions. The subsequent rise to 1.87% in the first five months of 2025 likely marks the onset of a new—and potentially intensified—epidemic cycle, driven by this pathogen's intrinsic periodicity and a post-pandemic “immunity debt” in the pediatric population.

In addition to its multi-year cyclical pattern, CPP demonstrates distinct seasonal clustering that varies geographically. Studies from France and South Korea reported a predominance of cases from late summer to autumn (July–December) (2, 10), whereas a spring peak (February–April) was observed in the United States (15). A study from Wuxi, China, identified the peak period of CP infections as occurring from November to February of the following year (16). Consistent with this cool-season pattern, our study found that pediatric CPP cases peaked during winter and spring. These divergent seasonal trends across regions strongly suggest that local climate and environmental conditions—such as ambient temperature, humidity, and associated human behavior—play a key role in modulating the timing of outbreaks. Furthermore, the seasonality observed in our cohort may also reflect broader immunological and behavioral shifts following the COVID-19 pandemic (5).

One study reported a broad age distribution for CPP, with children accounting for the majority (73%) of cases (2). The median age of pediatric CPP patients was 8 years (range: 2 months–15 years) (2), consistent with our findings (mean age 8.12 years, range: 1 month–16 years). In the present study, school-aged children (7–16 years) constituted the highest proportion of cases (68.04%), aligning with a Mexican study that also reported high CPP prevalence in this age group (3). This was followed by infants aged 1 month to 1 year (22.34%), suggesting potential regional variations in CPP epidemiology within the Shijiazhuang area that warrant the establishment of a long-term surveillance system. Regarding sex distribution, 64.30% of the cases were male. Similarly, Edouard et al. (2) reported a male predominance (51%; 19/37 cases). The influence of sex on susceptibility to CP infection requires further investigation. In this study, pediatric CPP patients had a mean hospitalization duration of 7.52 days and a total mean illness duration of 14.81 days. These findings are consistent with previously reported hospitalization durations [6.7 days (10)] and total illness durations [17 days (17)]. The relatively prolonged disease course imposes considerable burdens on patients, families, and healthcare systems.

Pediatric CPP typically presents with a subacute onset, with persistent cough (100%) and fever (88%–100%; median thermal peak: 38.80 °C) as hallmark clinical features (4). Other manifestations may include expectoration (30%–70%), chest pain (40%), dyspnea, hemoptysis, and hypoxia. Pulmonary auscultation frequently reveals wet rales (3, 17, 18). Consistent with previous studies, cough was the most common symptom in our study (98.97%), followed by fever (45.70%; median peak: 38.44 °C), while pulmonary rales were detected in 48.11% of patients (140/291).

Pediatric patients with CPP often exhibit a marked systemic inflammatory response. Previous studies have reported elevated inflammatory markers—such as neutrophil percentage (median: 69%), C-reactive protein (median: 10.21 mg/L), procalcitonin, and lactate dehydrogenase—as well as coagulation abnormalities and hepatic dysfunction in a subset of patients (17, 18). Consistent with these reports, our study also demonstrated increased white blood cell counts and elevated inflammatory markers, including C-reactive protein. Additionally, we observed coagulation disturbances (e.g., elevated D-dimer), cardiac enzyme abnormalities, hepatic impairment, and immune dysregulation. Notably, however, our analysis did not identify any distinctive clinical features among children who developed these transient laboratory abnormalities. The absence of specific clinical correlates, together with follow-up data showing complete resolution of these abnormalities after discharge, suggests that they likely reflect a nonspecific, transient reaction to acute infection rather than a distinct clinical subtype.

Animal studies have shown that CP infection can disseminate to multiple organ systems following intranasal transmission (9), explaining how CP infection may induce multisystem pathology beyond pulmonary involvement. Such systemic involvement may include immunosuppression, pleural effusion, pulmonary embolism, and dermatological manifestations (e.g., urticaria, erythema nodosum, erythema multiforme, IgA vasculitis, acute generalized exanthematous pustulosis) (17, 19, 20, 21). These observations reinforce the systemic inflammatory response and organ involvement mechanisms associated with CP infection. In a recent severe case, Vergara et al. (22) reported a 24-year-old patient with CP infection who developed mucocutaneous respiratory syndrome. These findings highlight the importance of vigilant monitoring for systemic complications in pediatric CPP patients.

CP infections can present as either single or mixed infections, with regional differences observed in pathogen detection rates among pediatric CPP patients. A South Korean study reported that 81.0% (17/21) of CPP cases involved multiple pathogens, including coinfection with *Haemophilus influenzae* in 62% (13/21) and *Streptococcus pneumoniae* (SP) in 29% (6/21) (10). Mexican investigators found that 64% (16/25) of pediatric CPP patients had mycoplasma coinfection (3), while a 2025 Chinese study identified one case (10%) with concurrent *Mycoplasma pneumoniae* and Bordetella pertussis infection (16). In our study, 60.82% of the patients (177/291) had mixed infections. The most frequently detected co-pathogens were *Rhinovirus* (58/291, 19.93%), *Haemophilus influenzae* (53/291, 18.21%), and SP (53/291, 18.21%), with the dominant pathogens varying by age group. Notably, coinfections of CP with other respiratory pathogens may exacerbate lung injury and immune dysregulation, posing considerable treatment challenges (17). These findings highlight the need for ongoing regional surveillance of CP prevalence patterns.

Lung imaging characteristics in children with CPP are often nonspecific. Previous studies have indicated that more than half of pediatric patients present with unilateral pneumonia, and a minority show bilateral lung involvement (16, 17). Subpleural masses with high-density shadows accompanied by peripheral halo signs may have diagnostic value, as observed in 60% of cases (6/10) in one study (16). Air bronchogram signs were present in 70% (7/10), whereas pulmonary embolism was noted in 20% (2/10) of the children with CPP (16). A small proportion also exhibited patchy pulmonary consolidation (16). In our study, over half of the patients had involvement of two or more lung lobes, with some showing halo signs, air bronchogram signs, or pulmonary consolidation. Consistent with previous reports, no pathognomonic imaging features specific to CPP were identified.

Effective management of pediatric CPP requires early and accurate diagnosis. Even in immunocompetent patients, delayed identification of CP infection may lead to rapidly progressive pulmonary damage (11). Diagnostic challenges arise from the unique etiological characteristics of CP: although cell culture remains the historical gold standard, it is technically demanding and exhibits low sensitivity in routine diagnostic settings, while serological assays are susceptible to cross-reactivity. QPCR technology, while a detection limit of 50–100 femtograms and 100% specificity, is currently recommended for the diagnosis of CP infection (3). Compared with conventional pathogen detection methods, tNGS enables broader pathogen coverage, offers higher sensitivity and faster turnaround time, and maintains superior diagnostic accuracy (23). In this study, the combined use of qPCR and tNGS enabled rapid and accurate diagnosis of pediatric CPP patients. Concurrently, bronchoscopy with BAL provides significant diagnostic collaboration (24). Bronchoscopic examination in our cohort revealed bronchial mucositis with flocculent purulent secretions. BAL fluid analysis showed a predominance of neutrophils—a shift from the normal alveolar macrophage profile—supporting the characteristic inflammatory response associated with CP infection. Although diagnostic technologies continue to advance, accurate pathogen identification still requires the integration of clinical evaluation, imaging findings, and molecular biology results. This comprehensive approach remains essential for accurate diagnosis and optimized management of CPP.

CP is an obligate intracellular bacterium characterized by a unique biphasic developmental cycle, alternating between the infectious elementary body and the replicative reticulate body (5). This biological strategy enables efficient intracellular replication while facilitating transmission to new host cells (25, 26). The intracellular localization of CP shields it from many antimicrobials, underscoring the importance of selecting antibiotics with adequate tissue penetration. Current guidelines recommend that antibacterial therapy for CPP should not exceed 21 days (27). Azithromycin remains the preferred initial agent due to its shorter course and favorable safety profile (28). Omadacycline, a novel tetracycline-derived agent, demonstrates promising efficacy against quinolone-resistant CP strains (24) and represents a valuable alternative in the context of increasing CP resistance (29). It is particularly indicated for patients with moxifloxacin resistance or those at risk for QT-interval prolongation (30). In our study, doxycycline was the most frequently prescribed antibiotic, followed by azithromycin and erythromycin—a prescribing pattern that may reflect the older age distribution of our pediatric patients. Among the seven patients who required an in-hospital antibiotic change, two were switched to azithromycin due to severe gastrointestinal intolerance to initial macrolide therapy (erythromycin or clarithromycin). The other five patients were transitioned to a tetracycline agent (minocycline or doxycycline) after showing no clinical improvement following three days of azithromycin.

Adjunctive corticosteroid therapy has shown potential benefit in severe or organized CP-associated pneumonia (10, 18). However, a South Korean study observed no significant differences in clinical outcomes—including age, sex, duration of hospitalization, time to fever resolution, or chest imaging findings—between steroid-treated (*n* = 16) and untreated (*n* = 5) groups (10). The therapeutic role of glucocorticoids in CPP, including specific indications for their use, warrants further systematic investigation.

This study has several limitations. This study represents a single-center retrospective analysis with a relatively limited sample size. Future investigations should prioritize large-scale, multicenter studies to enable more robust validation. Additionally, several critical areas require further exploration, such as long-term prognostic monitoring in pediatric CP, the clinical implications of pathogen co-detection, and the optimization of pathogen detection methodologies.

## 5 Conclusion

In summary, our study demonstrates that CPP is challenging to diagnose due to its non-specific clinical symptoms. Clinicians should therefore maintain a high index of suspicion for CPP during seasonal outbreaks, including in children with atypical symptoms. Our findings support the combined use of qPCR and tNGS as a valuable diagnostic strategy to facilitate targeted antimicrobial therapy. Future efforts should focus on integrating these advanced diagnostic modalities into standardized clinical pathways to improve patient outcomes.

## Data Availability

The original contributions presented in the study are included in the article/Supplementary Material, further inquiries can be directed to the corresponding author.
