# Supplementary material for: Epidemiological and clinical analysis of 291 children diagnosed with Chlamydia pneumoniae pneumonia: a 10-year retrospective study in Shijiazhuang, China
Source: Front Pediatr. 2025 Oct 24;13:1681564. doi: 10.3389/fped.2025.1681564 (PMC12592089; doi:10.3389/fped.2025.1681564)
Supplement: Supplementary file 1 [file Presentation1.pptx]

## Slide 1
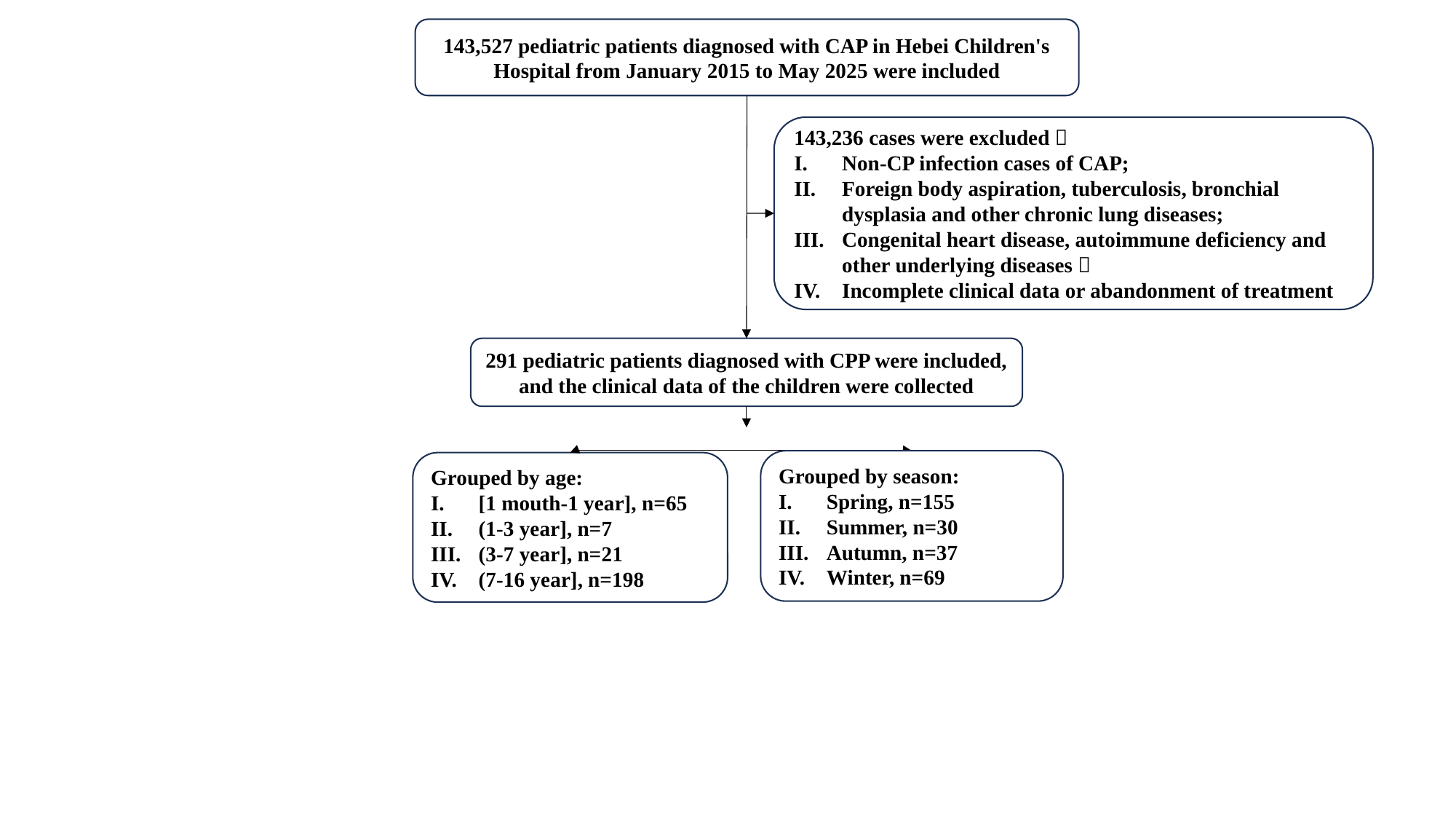

143,527 pediatric patients diagnosed with CAP in Hebei Children's Hospital from January 2015 to May 2025 were included
143,236 cases were excluded：
Non-CP infection cases of CAP;
Foreign body aspiration, tuberculosis, bronchial dysplasia and other chronic lung diseases;
Congenital heart disease, autoimmune deficiency and other underlying diseases；
Incomplete clinical data or abandonment of treatment
291 pediatric patients diagnosed with CPP were included, and the clinical data of the children were collected
Grouped by season:
Spring, n=155
Summer, n=30
Autumn, n=37
Winter, n=69
Grouped by age:
[1 mouth-1 year], n=65
(1-3 year], n=7
(3-7 year], n=21
(7-16 year], n=198
