# Supplementary material for: Epidemiological and clinical analysis of 291 children diagnosed with Chlamydia pneumoniae pneumonia: a 10-year retrospective study in Shijiazhuang, China
Source: Front Pediatr. 2025 Oct 24;13:1681564. doi: 10.3389/fped.2025.1681564 (PMC12592089; doi:10.3389/fped.2025.1681564)
Supplement: Supplementary file 2 [file Datasheet1.pdf]

(i) Immune Function Panel

Test	Item
Immunological Parameters	IgA, IgD, IgM, IgG, IgE;<br>IL-1β, IL-2, IL-4, IL-5, IL-6, IL-8, IL-10, IL-17A, IL-12p70;<br>TNF-α;<br>IFN-α, IFN-γ;
T Lymphocyte Subsets	Total T Cells, Helper T Cells, Suppressor T Cells, Helper/Suppressor Ratio, Double-Negative T Cells, Double-Positive T Cells, Total B Cells, Natural Killer Cells, Total T Cell Count, Helper T Cell Count, Suppressor T Cell Count, Total B Cell Count, Natural Killer Cell Count.

(ii) Liver Function Panel

Test Item	Abbreviation	Result	Normal Range	Unit
Total Bilirubin	TBil	8.6	3 - 20	μmol/L
Direct Bilirubin	DBil	2.0	1.7 - 6.8	μmol/L
Alanine Aminotransferase	ALT	22	8 - 42	U/L
Aspartate Aminotransferase	AST	41	22 - 59	U/L
Total Bile Acids	TBA	3.0	0.5 - 10	μmol/L
Gamma-Glutamyl Transferase	GGT	12	5 - 19	U/L

(iii) Renal Function Panel

Test Item	Abbreviation	Result	Normal Range	Unit
Urea	Urea	3.98	2.3 - 6.7	mmol/L
Creatinine (Enzymatic)	Cr	22	13 - 33	μmol/L
Uric Acid	UA	207	208 - 428	μmol/L

(iv) Cardiac Enzyme Panel

Test Item	Abbreviation	Result	Normal Range	Unit
Creatine Kinase	CK	91	50 - 310	U/L
Lactate Dehydrogenase	LDH	345	109 - 245	U/L
Alpha-Hydroxybutyrate Dehydrogenase	α-HBDH	282	72 - 182	U/L
Creatine Kinase Isoenzymes	CK-MB	43.00	< 5.0	μg/L
