# Supplementary material for: Epidemiological and clinical analysis of 291 children diagnosed with Chlamydia pneumoniae pneumonia: a 10-year retrospective study in Shijiazhuang, China
Source: Front Pediatr. 2025 Oct 24;13:1681564. doi: 10.3389/fped.2025.1681564 (PMC12592089; doi:10.3389/fped.2025.1681564)
Supplement: Supplementary file 3 [file Datasheet2.pdf]

The methods for NGS analysis (Library preparation, sequencing, and bioinformatics analysis):

An unique genomic region spanning around 150 bp was targeted and amplified for CPP detection at species level.
Both DNA and RNA were extracted from the BALF. The library preparation included: RNA reverse transcription and first strand cDNA synthesis, multiplex PCR amplification, adaptor ligation and index amplification. After pooling, denaturation cycling, and DNA nanoball (DNB) preparation, the libraries were sequenced using the MGISEQ-2000 sequencing platform. The bioinformatics pipeline was then applied including the following stages: quality control (total reads, Q30, internal control), low quality reads filtering, reads alignment, and background filtering. A total of 232 pathogens, 31 drug-resistant genes, and 11 virulence factors were targeted for detection. The final estimated abundance were reported as normalized reads assigned to the genus and/or species per million.
