# Supplementary material for: Epidemiological and clinical analysis of 291 children diagnosed with Chlamydia pneumoniae pneumonia: a 10-year retrospective study in Shijiazhuang, China
Source: Front Pediatr. 2025 Oct 24;13:1681564. doi: 10.3389/fped.2025.1681564 (PMC12592089; doi:10.3389/fped.2025.1681564)
Supplement: Supplementary file 4 [file Datasheet3.pdf]

Supplementary table 1：

Respiratory Pathogen qPCR 13-Target Panel
Influenza A virus, Influenza A virus H1N1, Influenza A virus H3N2, Influenza B virus, Parainfluenza virus, Adenovirus, Bocavirus, Rhinovirus, Metapneumovirus, Respiratory syncytial virus, Coronavirus, Mycoplasma pneumoniae, Chlamydia pneumoniae

Supplementary table 2：

tNGS (Targeted Next-Generation Sequencing), 274 Targets Associated with Respiratory Infection Syndromes		
Type		Target Name
Bacterial Pathogens (72 species)	Gram-Positive Bacteria (34 species)	Streptococcus pneumoniae, Streptococcus pyogenes, Streptococcus agalactiae, Streptococcus constellatus, Streptococcus dysgalactiae, Staphylococcus aureus, Mycobacterium tuberculosis complex, Mycobacterium avium complex, Mycobacterium kansasii, Nocardia abscessus, Nocardia otitidiscaviarum, Nocardia aciditolerans, Enterococcus faecalis, Enterococcus faecium, Corynebacterium diphtheriae, Corynebacterium striatum, Rhodococcus equi, Listeria monocytogenes, Erysipelothrix rhusiopathiae, Clostridium perfringens, Bacillus anthracis, Nocardia brasiliensis, Nocardia asteroides, Nocardia wallacei, Nocardia carnea, Nocardia paucivorans, Nocardia pseudobrasiliensis, Nocardia veterana, Nocardia hollensis, Nocardia concava, Nocardia farcinica, Tropheryma whipplei
	Gram-Negative Bacteria (38 species)	Yersinia pestis, Pseudomonas aeruginosa, Haemophilus influenzae, Legionella pneumophila, Legionella bozemanii, Legionella micdadei, Klebsiella pneumoniae, Klebsiella aerogenes, Klebsiella oxytoca, Acinetobacter baumannii, Escherichia coli, Enterobacter cloacae complex, Proteus mirabilis, Proteus vulgaris, Proteus penneri, Streptobacillus moniliformis, Bordetella pertussis, Bordetella parapertussis, Bordetella avium, Bordetella bronchiseptica, Bordetella holmesii, Burkholderia mallei, Burkholderia pseudomallei, Burkholderia cepacia, Stenotrophomonas maltophilia, Morganella morganii, Salmonella enterica, Pasteurella multocida, Neisseria meningitidis, Moraxella catarrhalis, Bacteroides fragilis, Serratia marcescens, Fluorescentibacter dumosii, Brucella spp., Francisella tularensis, Citrobacter freundii, Elizabethkingia meningoseptica, Achromobacter

		xylosoxidans
Viral Pathogens (95 species)	DNA Viruses (33 species)	Monkeypox virus, Human bocavirus types 1-4, Human adenovirus (Mastadenovirus), Human mastadenovirus A, Human mastadenovirus B, Human adenovirus B3, Human adenovirus 7, Human adenovirus 11, Human adenovirus 14, Human adenovirus 21, Human adenovirus 34, Human adenovirus 35, Human mastadenovirus C, Human adenovirus 1, Human adenovirus 2, Human adenovirus 5, Human adenovirus 6, Human mastadenovirus D, Human adenovirus 56, Human mastadenovirus E, Human adenovirus E4, Human alphaherpesvirus 1 (HSV-1), Human alphaherpesvirus 2 (HSV-2), Human alphaherpesvirus 3 (VZV), Human betaherpesvirus 5 (CMV), Human betaherpesvirus 6A, Human betaherpesvirus 6B, Human gammaherpesvirus 4 (EBV), BK polyomavirus, JC polyomavirus
	RNA Viruses (62 species)	Influenza A virus, Influenza A (H1N1), Influenza A (H3N2), Influenza A (H5N1), Influenza A (H5N6), Influenza A (H7N9), Influenza B virus, Influenza C virus, Human metapneumovirus, Human respiratory syncytial virus A, Human respiratory syncytial virus B, Human coronavirus OC43, Human coronavirus 229E, Human coronavirus HKU1, Human coronavirus NL63, SARS-CoV-2, Middle East respiratory syndrome coronavirus, Mumps virus, Human respirovirus 1 (Parainfluenza 1), Human rubulavirus 2 (Parainfluenza 2), Human respirovirus 3 (Parainfluenza 3), Human rubulavirus 4 (Parainfluenza 4), Measles virus, Human metapneumovirus, Human rhinovirus, Rhinovirus A, Rhinovirus B, Rhinovirus C, Enterovirus, Enterovirus A, Enterovirus A71, Enterovirus B, Enterovirus C, Enterovirus D, Enterovirus D68, Coxsackievirus A2, Coxsackievirus A5, Coxsackievirus A6, Coxsackievirus A8, Coxsackievirus A9, Coxsackievirus A10, Coxsackievirus A12, Coxsackievirus A16, Coxsackievirus A19, Coxsackievirus B1, Coxsackievirus B2, Coxsackievirus B3, Coxsackievirus B4, Coxsackievirus B5, Coxsackievirus B6, Echovirus E4, Echovirus E11, Echovirus E17, Echovirus E18, Echovirus E19, Echovirus E20, Echovirus E24, Echovirus E25, Echovirus E33, Poliovirus 1, Parechovirus A, Rubella virus
Fungal Pathogens (50 species)		Candida albicans, Candida parapsilosis, Candida metapsilosis, Candida glabrata, Candida tropicalis, Pichia kudriavzevii (Candida krusei), Cryptococcus neoformans, Cryptococcus gattii, Cryptococcus spp. (including C. deuterogattii, C. bacillisporus, C. tetragattii), Histoplasma capsulatum, Talaromyces marneffei, Aspergillus niger, A. fumigatus, A. flavus, A. tardus, A. nidulans, A. oryzae, A. terreus, Lichtheimia ramosa, Lichtheimia corymbifera, Rhizopus oryzae (R. arrhizus), Rhizopus microsporus, Mucor circinelloides, Mucor racemosus, Mucor irregularis, Mucor ramosissimus, Pneumocystis jirovecii, Coccidioides posadasii, Coccidioides immitis, Trichosporon

	asahii, Trichosporon inkin, Scedosporium apiospermum, Sporothrix brasiliensis, Sporothrix schenckii, Syncephalastrum monosporum, Syncephalastrum racemosum, Lomentospora prolificans, Paracoccidioides brasiliensis, Paracoccidioides lutzii, Microascus trigonosporus, Microascus albofasciatus, Fusarium solani, Geotrichum candidum, Sporothrix mexicana, Sporothrix globosa<br>Other Pathogens (15 species):<br>Mycoplasma pneumoniae, Chlamydia pneumoniae, Chlamydia psittaci, Chlamydia trachomatis, Bartonella bacilliformis, Bartonella henselae, Bartonella quintana, Rickettsia rickettsii, R. prowazekii, R. typhi, Orientia tsutsugamushi, Coxiella burnetii, Mycoplasma hominis, Ureaplasma urealyticum, Paragonimus westermani
Other Pathogens (15 species)	Mycoplasma pneumoniae, Chlamydia pneumoniae, Chlamydia psittaci, Chlamydia trachomatis, Bartonella bacilliformis, Bartonella henselae, Bartonella quintana, Rickettsia rickettsii, R. prowazekii, R. typhi, Orientia tsutsugamushi, Coxiella burnetii, Mycoplasma hominis, Ureaplasma urealyticum, Paragonimus westermani
Drug Resistance Genes (31)	IMP、NDM、SPM、KPC、PER、VIM、SIM、DIM、OXA、ACT、GIM、CTX-M、GES、VEB、CMY、OKP、TLA、ACC、MOX、MIR、QnrA、QnrB、mecA、MCR、vanA、<br>vanB、vanC、SHV、FOX、sul、23S rRNA
Drug Resistance Loci (4)	Mycoplasma pneumoniae 23S rRNA gene (positions 2063, 2064, 2067, 2617)
Virulence Genes (11)	rmpA、fyuA、fepA、iroN、iutA、cnf1、sat、eta、etb、tox、MPN372
